# Assay-Dependent Variability of Antioxidant Responses in Hop Extracts: Implications for Cross-Study Comparability and Antioxidant Interpretation

**DOI:** 10.3390/molecules31122066

**Published:** 2026-06-12

**Authors:** Nora Haring, Blažena Drábová, Želmíra Balážová, Altynay Burkhatovna Abuova, Milan Chňapek

**Affiliations:** 1 Faculty of Biotechnology and Food Sciences, Slovak University of Agriculture in Nitra, Tr. A. Hlinku 2, 949 76 Nitra, Slovakia; qharing@uniag.sk (N.H.); zelmira.balazova@uniag.sk (Ž.B.); 2 Department of Engineering and Technology of Food Production, International University of Engineering and Technology, Almaty 050000, Kazakhstan

**Keywords:** *Humulus lupulus*, antioxidant capacity, assay-dependent response, extraction selectivity, polyphenols, chemometrics

## Abstract

Antioxidant activity of plant extracts is commonly interpreted as a directly comparable property despite substantial methodological differences among analytical assays and extraction systems. This study investigated how extraction selectivity and assay chemistry influence antioxidant-associated responses in hop (*Humulus lupulus* L.) extracts by integrating experimental and literature-derived datasets. Extracts obtained using different solvents and extraction techniques were evaluated using ABTS, DPPH, and Folin–Ciocalteu (TPC) systems. Multivariate statistical analyses, including principal component analysis (PCA), correlation analysis, and non-parametric comparisons, were applied to normalized datasets to assess assay-dependent variability and cross-study comparability. The results suggested substantial divergence between ABTS- and DPPH-associated responses, including a statistically significant negative correlation between both assay systems. PCA indicated assay-selective separation patterns, while TPC values did not consistently correlate with antioxidant-associated responses. Different extraction conditions were associated with distinct antioxidant response profiles, suggesting selective redistribution of analytically detectable antioxidant fractions rather than uniform changes in antioxidant capacity. Based on these observations, this study proposes the Assay–Extraction Interaction Framework (AEIF), an interpretative framework that views antioxidant activity as a context-dependent analytical response rather than a universal intrinsic property of the extract.

## 1. Introduction

Hop (*Humulus lupulus* L.) represents a chemically complex plant matrix rich in biologically active compounds, including phenolic acids, flavonoids, prenylated flavonoids, and α-bitter acids, which substantially contribute to the antioxidant-associated properties of hop extracts and beer products. Owing to this diverse phytochemical composition, hop has attracted increasing interest in antioxidant research, nutraceutical science, and functional food applications [1,2,3]. Unlike simplified model antioxidant systems, however, hop extracts constitute chemically heterogeneous matrices containing compounds with markedly different polarity, redox behavior, stability, and reaction kinetics.

Despite this complexity, antioxidant activity in plant extracts is still frequently interpreted throughout the literature as a relatively universal and directly comparable property of the extract, commonly represented by a single numerical value obtained using one analytical assay. However, antioxidant assays such as DPPH, ABTS, and Folin–Ciocalteu total phenolic content (TPC) determination rely on fundamentally different reaction mechanisms and exhibit different selectivity toward individual antioxidant fractions [2,4,5]. Consequently, different assay systems may capture distinct dimensions of antioxidant behavior rather than describe a single universally transferable antioxidant property.

This analytical divergence becomes particularly important in complex plant extracts because extraction conditions strongly influence extract composition itself. Solvent polarity, extraction temperature, pressure, and extraction intensity may selectively enrich structurally different phytochemical groups, thereby reshaping the relative abundance of analytically detectable antioxidant fractions [6,7,8]. At the same time, antioxidant datasets are commonly reported using analytically heterogeneous formats, including IC50 values, percentage inhibition, Trolox equivalent antioxidant capacity (TEAC), gallic acid equivalents (GAE), or ferulic acid equivalents (FAE), while extraction procedures, assay conditions, and calibration approaches frequently differ among studies. Consequently, part of the variability observed throughout antioxidant literature may reflect extraction-driven analytical heterogeneity and assay-associated divergence rather than true biological differences among analyzed materials.

Most currently available studies investigating hop antioxidant activity remain primarily focused on extraction optimization and identification of extraction conditions producing the highest antioxidant or TPC values [6,7,9]. Such approaches implicitly assume that higher assay responses directly correspond to higher antioxidant potential of the extract itself. However, in chemically heterogeneous systems, different extraction strategies may selectively enrich distinct phytochemical fractions, while different assay systems preferentially respond to different antioxidant mechanisms and compound classes. Antioxidant responses may therefore represent context-dependent analytical outcomes shaped by the interaction between extraction chemistry and assay selectivity rather than universally transferable measures of antioxidant potential.

**Figure 1:**
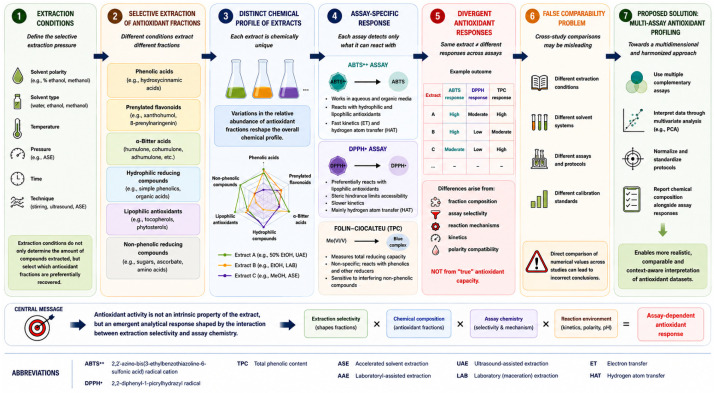
Proposed Assay–Extraction Interaction Framework (AEIF) illustrating how extraction conditions selectively reshape antioxidant fractions and generate assay-dependent antioxidant responses across ABTS, DPPH, and TPC systems. The framework highlights the context-dependent nature of antioxidant activity and the resulting limitations in direct cross-study comparability of antioxidant datasets.

To the best of our knowledge, no previous study has systematically integrated extraction selectivity, assay chemistry, dataset harmonization, and multivariate interpretation into a unified interpretative framework describing assay-dependent antioxidant behavior in hop extracts. Therefore, the present study proposes the Assay–Extraction Interaction Framework (AEIF), which interprets antioxidant activity as a context-dependent assay-associated response shaped by the interaction between extraction chemistry, extract composition, assay selectivity, and reaction environment (Figure 1). Within this context, the present study aimed to systematically investigate how extraction technique, solvent polarity, and extraction conditions influence antioxidant-associated responses in hop extracts, while evaluating the extent to which ABTS-, DPPH-associated responses and total phenolic content (TPC) provide convergent or divergent analytical interpretations within chemically heterogeneous matrices. Particular emphasis was placed on understanding the interplay between extraction chemistry and assay selectivity as a determinant of assay-dependent analytical outcomes.

The objective of this study was not to identify the ‘best’ extraction strategy or maximize antioxidant activity values, but rather to systematically evaluate how extraction selectivity and assay chemistry collectively shape antioxidant-associated responses and influence interpretation of heterogeneous antioxidant datasets. To address this objective, experimentally generated and literature-derived datasets encompassing ABTS-, DPPH-, and TPC-associated responses obtained under different extraction conditions and solvent systems were harmonized and evaluated using normalization strategies, principal component analysis (PCA), Spearman correlation analysis, and Kruskal–Wallis testing. Rather than introducing another comparative extraction study, the present work proposes a broader interpretative framework for evaluation of antioxidant activity in chemically heterogeneous plant extract systems.

## 2. Results

### 2.1. Dataset Heterogeneity Reflects Context-Dependent Antioxidant Responses

Descriptive analysis of the normalized datasets revealed substantial variability among extraction techniques, solvents, and applied antioxidant assay systems. Distinct assay-associated response patterns were observed across extraction conditions. Ultrasound-assisted extraction was frequently associated with elevated DPPH-associated responses within parts of the harmonized dataset whereas comparatively higher ABTS-associated responses were observed in extracts prepared using ASE 350. Methanolic extracts tended to exhibit higher DPPH-associated responses, whereas ethanolic extracts were more frequently associated with elevated ABTS responses and higher TPC values.

The observed variability among assay systems suggests that antioxidant-associated responses were shaped by the interaction between extraction conditions and analytical methodology. Notably, extracts exhibiting elevated responses in one assay system did not consistently demonstrate similarly increased responses across other analytical platforms, indicating assay-associated divergence in antioxidant response behavior.

Because the harmonized dataset combined experimentally generated and literature-derived observations obtained under heterogeneous analytical conditions, including differences in extraction procedures, solvent systems, assay protocols, and reporting formats, the observed response patterns should be interpreted as descriptive and exploratory rather than universally transferable relationships. Consequently, the present analysis aimed to identify assay-associated trends across heterogeneous datasets rather than establish direct causal relationships between extraction conditions and antioxidant responses.

The Shapiro–Wilk test demonstrated that several datasets did not follow a normal distribution, particularly within subsets categorized according to extraction conditions and solvent type. Based on these findings, non-parametric statistical methods were subsequently applied for further analyses.

### 2.2. Principal Component Analysis Indicates Assay-Associated Variability

Principal component analysis (PCA) was performed on normalized ABTS-, DPPH-associated response, and total phenolic content (TPC) datasets (*n* = 27) to explore multivariate relationships among antioxidant-associated parameters and visualize assay-associated variability across the investigated extracts. Because the dataset integrated experimentally generated and literature-derived observations obtained under heterogeneous analytical conditions, PCA was interpreted as an exploratory multivariate visualization tool intended to identify broad response patterns and covariance structures rather than infer direct causal relationships or establish definitive mechanistic interpretations. The PCA model explained 81.27% of the total variance within the first two principal components, with PC1 accounting for 54.09% and PC2 for 27.17% of the variance. The PCA model explained 81.27% of the total variance within the first two principal components, with PC1 accounting for 54.09% and PC2 for 27.17% of the variance.

The PCA biplot indicated separation between ABTS- and DPPH-associated responses within the principal component space, suggesting assay-associated variability in antioxidant response behavior across the analyzed datasets. ABTS exhibited a strong negative loading on the first principal component (−0.805), whereas DPPH showed a positive loading (0.772), indicating that extracts characterized by elevated responses in one assay system did not consistently exhibit similarly increased responses in the other. Opposing loading patterns observed for ABTS and DPPH may reflect differences in assay chemistry, reaction mechanisms, and compound selectivity rather than uniform antioxidant behavior across extraction systems. TPC displayed a weaker positive association with the first principal component (0.616), suggesting a less pronounced contribution to the observed assay-associated separation patterns.

The distribution of samples within the PCA space further illustrated substantial heterogeneity among the combined experimental and literature-derived datasets. Although partial distribution trends associated with extraction conditions were observed, PCA primarily highlighted assay-associated variability rather than distinct clustering according to extraction technique or solvent system. These findings suggest that differences in assay chemistry, reaction selectivity, and extraction context may collectively contribute to variability in antioxidant-associated responses.

**Figure 2:**
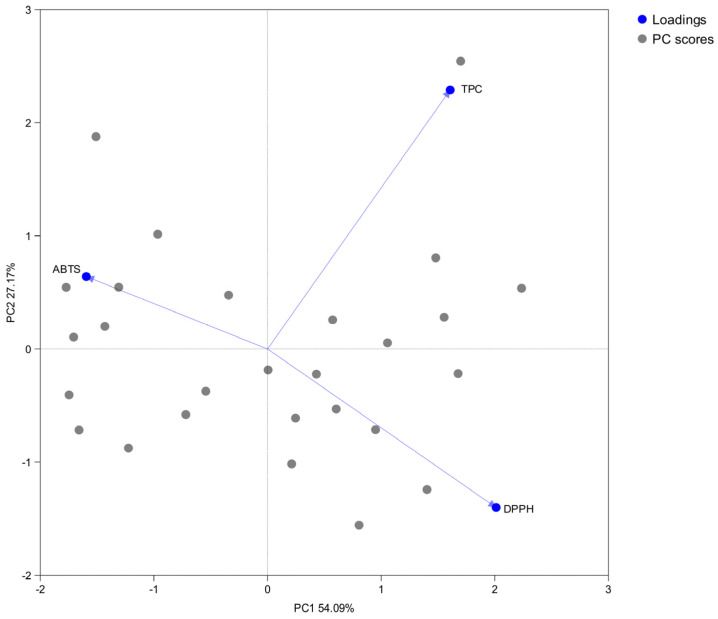
Principal component analysis (PCA) biplot of normalized ABTS-, DPPH-associated responses and total phenolic content (TPC) values across combined experimental and literature-derived datasets. Loading vectors illustrate the contribution and orientation of individual analytical systems within the principal component space, while grey points represent individual sample scores. The oppositely oriented loading vectors of ABTS- and DPPH-associated responses suggest assay-associated divergence in antioxidant response patterns. PC1 and PC2 explained 54.09% and 27.17% of the total variance, respectively. PCA is presented as an exploratory visualization of covariance patterns within the harmonized dataset (*n* = 27).

PCA was interpreted as an exploratory multivariate visualization tool intended to illustrate divergence patterns among antioxidant assay systems rather than establish direct mechanistic or causal relationships between extraction chemistry and antioxidant-associated responses (Figure 2).

### 2.3. ABTS- and DPPH-Associated Responses Indicate Assay-Associated Variability

Correlation analysis between normalized ABTS- and DPPH-associated responses across the harmonized dataset (*n* = 57) revealed a statistically significant negative correlation (Spearman’s ρ = −0.319, *p* = 0.015). The results suggested that extracts exhibiting elevated responses in one assay system did not consistently exhibit similarly increased responses in the other assay.

The scatter plot further illustrated substantial data dispersion and the absence of a consistent linear relationship between ABTS- and DPPH-associated responses. Several extracts characterized by elevated DPPH-associated responses exhibited only moderate or low ABTS-associated responses, whereas the opposite trend was observed in a subset of extracts. This pattern was observed across multiple extraction techniques and solvent systems within the harmonized dataset.

**Figure 3:**
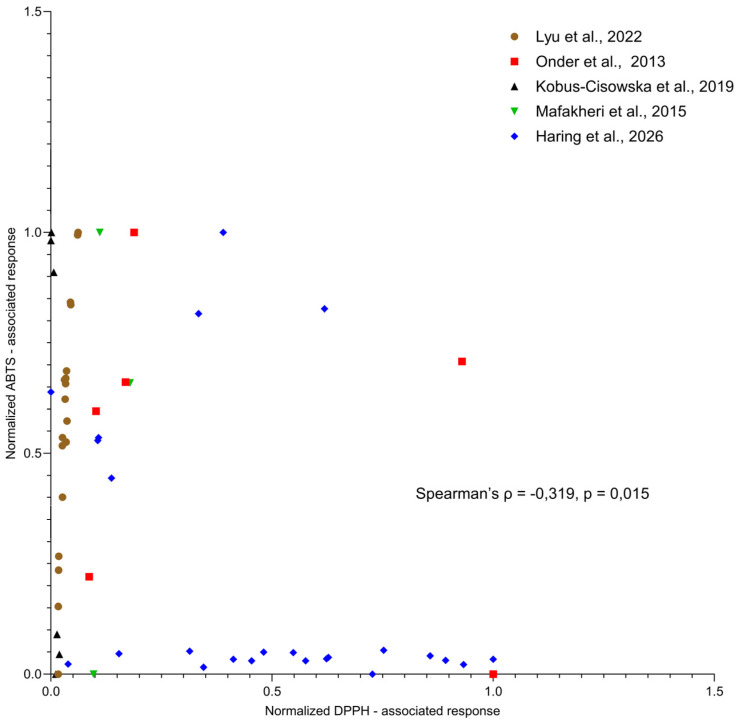
Scatter plot illustrating the relationship between normalized ABTS- and DPPH-associated responses across experimental and literature-derived hop extract datasets. Individual symbols represent independent datasets obtained from different studies and extraction conditions. The observed dispersion pattern and statistically significant negative correlation suggest assay-associated variability in antioxidant-associated responses within the harmonized dataset (*n* = 57). Literature-derived datasets included in the analysis were obtained from previously published studies [7,10,11,12,13].

The observed response patterns were further supported by PCA, in which ABTS and DPPH exhibited oppositely oriented loadings within the first principal component (Figure 3). Although the magnitude of the correlation was moderate, the combined multivariate and correlation analyses suggest assay-associated variability in antioxidant response behavior. These observations may reflect differences in assay chemistry, reaction mechanisms, compound selectivity, and polarity compatibility rather than uniform antioxidant behavior across extraction systems.

### 2.4. Total Phenolic Content Does Not Uniformly Predict Assay-Associated Antioxidant Responses

The relationship between total phenolic content (TPC) and assay-associated antioxidant responses was evaluated using correlation analysis on a matching dataset containing simultaneously available TPC, ABTS-, and DPPH-associated response values (*n* = 27) derived from the harmonized experimental and literature-based dataset. Only samples with simultaneously available normalized TPC and antioxidant response values were included in the analysis, whereas supporting FAE-based datasets were excluded from the primary statistical harmonization. The analysis suggested differing relationships between TPC and individual assay systems.

A weak positive correlation was observed between TPC and DPPH-associated responses (Spearman’s ρ = 0.252); however, this relationship was not statistically significant (*p* = 0.204). In contrast, a statistically significant negative correlation was identified between TPC and ABTS-associated responses (Spearman’s ρ = −0.444, *p* = 0.020).

The scatter plots further illustrated substantial variability within the analyzed dataset, as extracts characterized by comparable TPC values frequently exhibited markedly different ABTS- and DPPH-associated responses. This pattern was observed across multiple extraction techniques and solvent systems, particularly among ethanolic and methanolic extracts.

**Figure 4:**
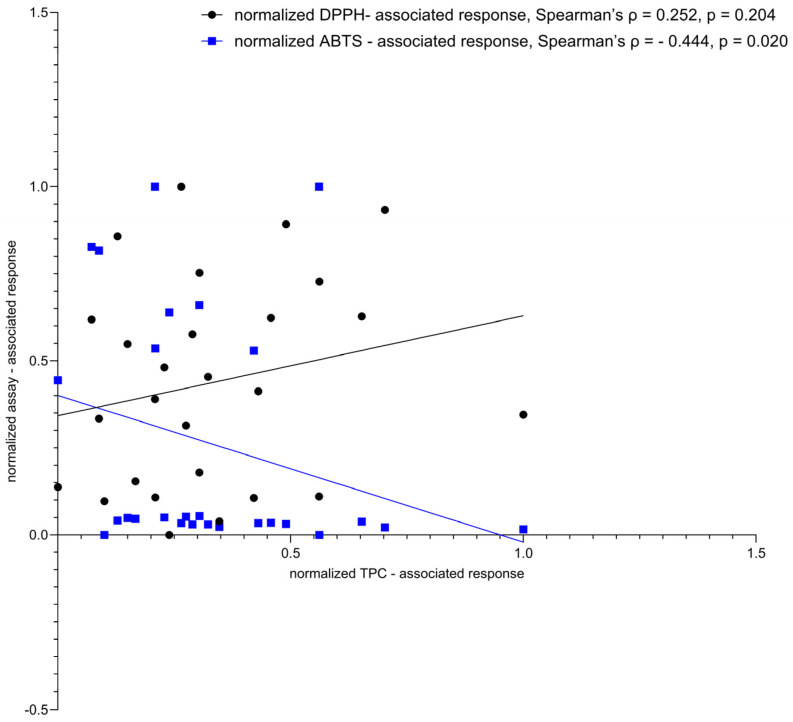
Correlation analysis between normalized total phenolic content (TPC) and assay-associated antioxidant responses determined using ABTS and DPPH systems across the harmonized experimental and literature-derived dataset (*n* = 27). Only samples with simultaneously available normalized TPC and antioxidant response values were included in the analysis, whereas supporting FAE-based datasets were excluded from the primary statistical harmonization. Regression lines illustrate differing relationships observed between TPC and individual assay systems, highlighting assay-associated variability in antioxidant-associated responses across the analyzed extracts.

Collectively, these findings suggest that the relationship between TPC and antioxidant-associated responses was not consistent across analytical systems and may be shaped by extraction context, assay selectivity, and differences in analytical response behavior rather than uniformly reflecting antioxidant-associated activity (Figure 4).

### 2.5. Extraction Conditions Reshape Assay-Dependent Antioxidant Response Profiles

**Figure 5:**
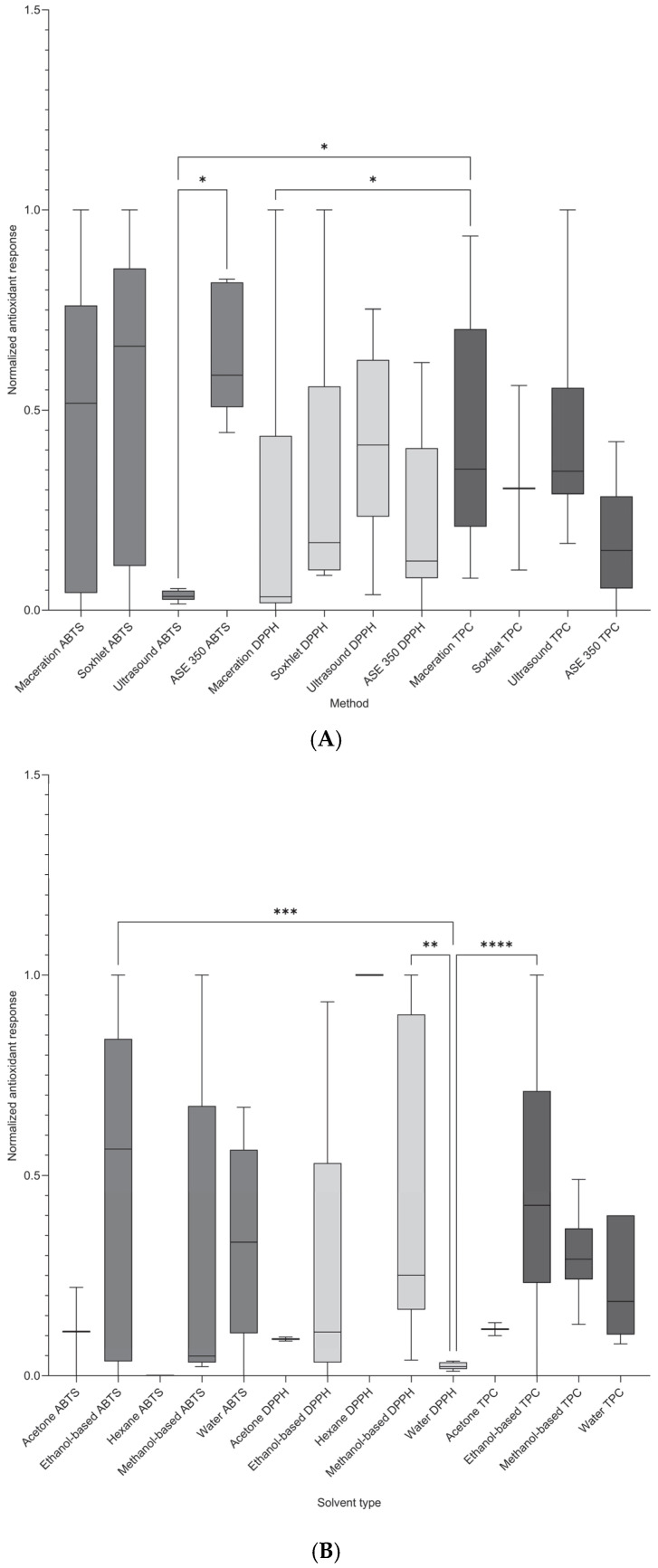
(**A**). Distribution of normalized ABTS-, DPPH-associated responses and total phenolic content (TPC) values according to extraction method (maceration, UAE, ASE 350) across the harmonized dataset. Observation numbers varied according to assay data availability. (**B**). Distribution of normalized ABTS-, DPPH-associated responses and total phenolic content (TPC) values according to extraction solvent category across the harmonized dataset. Boxplots illustrate median values, interquartile ranges, and data dispersion within solvent groups. Observation numbers varied according to data availability. Different boxplot shades indicate analytical systems: light gray = DPPH-associated response, medium gray = ABTS-associated response, and dark gray = total phenolic content (TPC). Asterisks indicate statistical significance levels: * *p* < 0.05, ** *p* < 0.01, *** *p* < 0.001, and **** *p* < 0.0001.

The relationship between extraction conditions and assay-associated antioxidant responses was evaluated using Kruskal–Wallis analysis across extraction categories with unequal observation numbers depending on assay availability. For extraction method comparison (Figure 5A), maceration datasets included *n* = 33 observations for ABTS- and DPPH-associated responses and *n* = 30 for TPC, whereas ultrasound-assisted extraction (UAE) and ASE datasets included *n* = 9 and *n* = 6 observations, respectively, across all assay systems.

For DPPH-associated responses, statistically significant differences among extraction categories were observed (χ^2^ = 10.6, *p* = 0.014), with several ultrasound-assisted extracts exhibiting comparatively elevated responses. Similarly, statistically significant differences among extraction categories were identified for ABTS-associated responses (χ^2^ = 10.0, *p* = 0.018), with extracts prepared using ASE 350 generally exhibiting higher responses compared with ultrasound-assisted extraction.

In contrast, no statistically significant differences in TPC values were observed among the evaluated extraction categories (χ^2^ = 6.73, *p* = 0.081), although ultrasound-assisted extraction and maceration displayed numerically higher TPC values.

For solvent category comparison (Figure 5B), observation numbers varied across solvent systems depending on dataset availability within the harmonized dataset. ABTS- and DPPH-associated response datasets were most frequently represented by methanolic and ethanolic extraction systems (*n* = 7–12), whereas several solvent categories included only one or two observations. TPC availability similarly varied among solvent systems due to incomplete reporting of simultaneously available antioxidant-associated responses across the included studies. Despite this heterogeneity, statistically significant differences among solvent categories were observed for DPPH-associated responses (χ^2^ = 20.8, *p* < 0.001), with comparatively elevated responses frequently associated with methanolic extracts. In contrast, no statistically significant solvent-associated differences were identified for ABTS-associated responses (χ^2^ = 5.45, *p* = 0.244). TPC values exhibited a solvent-associated trend; however, the effect did not reach statistical significance (χ^2^ = 7.36, *p* = 0.061).

The observed response patterns among the individual assay systems suggest that extraction conditions were not uniformly associated with all analytical systems. Because the harmonized dataset combined experimentally generated and literature-derived observations obtained under heterogeneous analytical conditions, these findings should be interpreted as descriptive assay-associated response patterns rather than direct evidence of causal extraction effects. Collectively, the results suggest that extraction context and assay selectivity may contribute to variability in antioxidant-associated response profiles across the analyzed extracts.

## 3. Discussion

### 3.1. Assay Divergence Reflects Selective Analytical Visibility Rather than Universal Antioxidant Capacity

The results of this study suggested divergence between ABTS- and DPPH-associated responses, which was reflected both in PCA and in the statistically significant negative correlation observed between the two assay systems. Although the magnitude of the correlation was moderate, the combined multivariate and correlation analyses suggest that individual antioxidant assays do not necessarily represent identical dimensions of antioxidant behavior, but rather may selectively respond to different antioxidant fractions present within chemically heterogeneous extracts. Similar inconsistencies between antioxidant assay systems have previously been reported in studies focused on hop-derived extracts and other complex plant matrices, where antioxidant rankings and measured antioxidant capacities varied substantially depending on the analytical system applied [3,14,15].

The observed divergence likely reflects the fundamentally different chemical principles underlying both assay systems. The DPPH assay employs a relatively sterically hindered and more lipophilic radical, whose reaction response is influenced by reaction medium polarity, antioxidant solubility, steric accessibility of reactive groups, and the availability of donor groups capable of participating in hydrogen atom transfer (HAT) mechanisms [16,17,18,19]. In addition, DPPH-based systems are known to exhibit sensitivity to solvent composition, reaction kinetics, and physicochemical compatibility between the radical system and extracted antioxidant fractions, which may influence antioxidant-associated responses in chemically complex plant extracts [3,20].

In contrast, the ABTS assay demonstrates broader compatibility with both hydrophilic and lipophilic antioxidants and is capable of proceeding through both electron transfer (ET) and hydrogen atom transfer mechanisms, thereby enabling analytical detection of a wider spectrum of antioxidant-active compounds [10,20]. Due to this broader analytical accessibility, ABTS-based systems may exhibit increased responsiveness toward structurally diverse phenolic fractions, including compounds that may remain partially undetected in DPPH-based systems because of steric or polarity-associated limitations [6,14].

These differences suggest that individual assay systems may not provide a universal representation of extract antioxidant capacity, but rather assay-selective analytical visibility of specific groups of compounds. Extracts exhibiting elevated DPPH-associated responses frequently did not display similarly increased ABTS responses, and vice versa, supporting the interpretation that individual assay systems may emphasize different chemical dimensions of antioxidant behavior. Importantly, this pattern was observed across multiple extraction techniques and solvent systems within the harmonized dataset, suggesting that assay divergence may reflect a broader characteristic of antioxidant-associated response behavior rather than variability among individual samples alone.

Comparable observations have also been discussed in previous studies focused on antioxidant assay chemistry, extraction selectivity, and polyphenol-rich plant extracts, where substantial differences in assay responsiveness were associated with solvent polarity, extract composition, and differing affinities of antioxidant compounds toward individual radical systems [21,22,23,24]. Collectively, these studies support the interpretation that antioxidant assays exhibit differing sensitivities toward specific antioxidant classes and that assay chemistry itself may shape interpretation of antioxidant-associated responses in chemically heterogeneous plant extracts.

The findings of the present study support the interpretation that antioxidant-associated responses in complex plant extracts should not be understood as a universal analytical parameter independent of the assay system used (Table 1). Instead, the obtained data suggest that antioxidant-associated responses may represent context-dependent analytical outcomes shaped by interactions among extraction selectivity, solvent polarity, extract chemical composition, and assay chemistry, which is consistent with the proposed Assay–Extraction Interaction Framework (AEIF). Although the present study does not directly identify the specific compounds responsible for the observed assay divergence, the combined analyses suggest that extraction conditions and assay chemistry may influence the analytical visibility of different antioxidant fractions, thereby contributing to distinct response patterns across analytical systems. Consequently, the objective of the present study was not compound-resolved mechanistic identification, but rather systematic evaluation of assay-dependent antioxidant response behavior across chemically heterogeneous extract systems.

Table 1 summarizes the principal mechanistic characteristics, analytical selectivity, and limitations of the ABTS, DPPH, and Folin–Ciocalteu (TPC) assay systems. The comparison highlights that individual assays selectively respond to different antioxidant fractions and reaction mechanisms, thereby contributing to assay-dependent variability in antioxidant-associated responses observed in complex hop extracts.

### 3.2. Solvent Selectivity Reshapes Detectable Antioxidant Fractions

The results of this study suggest that extraction solvents were associated not only with differences in the magnitude of assay-associated antioxidant responses, but also with differing analytical response patterns across assay systems. This effect was particularly evident in the distinct response profiles observed between the ABTS and DPPH systems across the analyzed solvents and extraction conditions. Similar solvent-dependent differences in antioxidant responsiveness have previously been reported in studies focused on hop extracts and other polyphenol-rich plant matrices, where extraction conditions influenced both extract composition and the resulting antioxidant response patterns [6,17].

Methanolic extracts frequently exhibited elevated DPPH-associated responses, whereas ethanolic extracts tended to display comparatively higher ABTS-associated responses together with higher TPC values. If antioxidant-associated responses represented a universal intrinsic property of the extract, a more consistent trend across analytical systems would be expected. Instead, differing or even opposing assay-associated responses were observed, suggesting that individual solvents may favor extraction of chemically distinct antioxidant fractions or alter their analytical detectability within individual assay systems. Comparable inconsistencies among antioxidant assays have also been observed in previous studies evaluating the influence of solvent systems on polyphenol extraction and antioxidant activity determination [14,15,21].

These observations are likely associated with differences in solvent polarity and extraction selectivity, which may influence the recovery of compounds differing in solubility, redox potential, molecular structure, and reaction kinetics. Previous studies have demonstrated that different extraction systems influence the abundance of flavonoids, phenolic acids, prenylated flavonoids, and α-bitter acids in hop extracts [10,16,17,20]. However, these compounds do not necessarily exhibit equivalent reactivity across individual assay systems, which may contribute to different analytical responses even among chemically comparable extracts [18,19].

Differences were also observed among extraction techniques. Ultrasound-assisted extraction frequently exhibited elevated DPPH-associated responses accompanied by comparatively lower ABTS-associated responses, whereas ASE 350 displayed the opposite trend. These observations suggest that extraction conditions may influence not only extraction efficiency, but also the relative analytical detectability of antioxidant fractions preferentially responding within specific assay systems. Elevated temperature and pressure during ASE extraction may facilitate recovery of more polar or less readily extractable compounds, whereas milder extraction conditions such as ultrasound-assisted extraction may preserve fractions exhibiting higher DPPH-associated responsiveness. Similar effects of extraction intensity and extraction conditions on antioxidant composition and assay responsiveness have also been discussed in studies focused on advanced extraction systems and plant-derived antioxidants [6,22,23].

Comparable solvent-dependent differences have also been discussed in previous studies focused on antioxidant extraction systems and polyphenol-rich plant extracts, where differences in antioxidant-associated responses were linked to solvent polarity, extraction selectivity, and assay chemistry [21,22,23,24]. Collectively, these studies support the interpretation that solvents and extraction conditions may influence not only the quantity of extracted antioxidants, but also which antioxidant fractions become analytically more detectable within specific assay systems.

The findings of this study therefore support the interpretation that solvent effects in antioxidant literature should not be reduced merely to “higher” or “lower” antioxidant activity. Instead, the obtained data suggest that solvents and extraction techniques may selectively redistribute antioxidant fractions or alter their analytical detectability within individual assay systems, thereby influencing interpretation of antioxidant-associated responses in chemically heterogeneous plant extracts.

### 3.3. Total Phenolic Content Represents a Reductive Chemical Environment Rather than a Universal Functional Antioxidant Marker

The results of this study suggested that the relationship between total phenolic content (TPC) and assay-associated antioxidant responses was not consistent across individual analytical systems. While only a weak positive correlation without statistical significance was observed between TPC and DPPH-associated responses, a statistically significant negative correlation was identified between TPC and ABTS-associated responses. These findings suggest that elevated TPC values do not necessarily result in increased assay-associated antioxidant responses and that interpretation of TPC as a universal marker of antioxidant capacity may be oversimplified in chemically heterogeneous plant extracts. Similar inconsistencies between TPC values and antioxidant assay responses have also been reported in previous studies focused on polyphenol-rich plant matrices and hop-derived extracts [14,15,20].

In antioxidant literature, TPC is frequently interpreted as a rapid indicator of the antioxidant potential of plant extracts, with higher values often associated with increased antioxidant activity. However, the Folin–Ciocalteu assay does not react selectively only with phenolic antioxidants, but rather with a broad spectrum of reducing substances, including amino acids, reducing sugars, ascorbate, sulfites, and other redox-active compounds [19,24,25,26]. Consequently, the resulting TPC response may not represent the quantity of antioxidants in a functional sense, but rather the overall reducing environment of the analyzed chemical system. Previous studies have similarly emphasized that Folin–Ciocalteu-derived values should be interpreted cautiously because the assay reflects overall reducing capacity rather than selective quantification of phenolic antioxidants alone [3,17].

This effect may be particularly relevant in complex plant extracts whose chemical composition is influenced by extraction conditions and solvent polarity. Different extraction strategies may selectively enrich extracts with compounds exhibiting elevated Folin responses without necessarily generating increased ABTS- or DPPH-associated responses. Previous studies have further demonstrated that different groups of phenolic compounds exhibit distinct redox activities, reaction kinetics, and assay-associated behaviors across individual antioxidant systems [14,15,18,20]. Consequently, chemically similar extracts may exhibit substantially different antioxidant-associated responses depending on extraction selectivity and the analytical system applied.

An additional source of variability among published studies is the use of different calibration standards. Although most studies report TPC as gallic acid equivalents (GAE), some published works employ alternative calibration systems, such as ferulic acid equivalents (FAE), catechin equivalents, or tannic acid equivalents, which may generate systematically different values and complicate direct comparability among datasets. For this reason, only GAE-based datasets were included in the primary statistical analysis in the present study, whereas FAE-derived data were interpreted qualitatively. Similar concerns regarding inter-study comparability of TPC datasets and methodological standardization have also been highlighted in previous antioxidant methodology studies [21,23].

The observed inconsistency between TPC and assay-associated antioxidant responses further suggests that individual analytical systems may not respond uniformly to identical antioxidant fractions. Extracts characterized by comparable TPC values exhibited markedly different ABTS- and DPPH-associated responses, supporting the interpretation that antioxidant-associated behavior in complex plant extracts may be shaped not only by the quantity of phenolic compounds, but also by their chemical structure, polarity, redox properties, reaction kinetics, and assay selectivity. Similar conclusions regarding assay-associated analytical visibility and differing responsiveness toward individual phenolic fractions have also been reported in studies focused on antioxidant chemistry and extraction selectivity [6,22].

The findings of this study therefore support the interpretation that TPC should not be regarded as an independent universal marker of antioxidant capacity without consideration of assay chemistry and extraction context. Instead, the obtained data suggest that TPC may represent a broader indicator of the reducing chemical environment of the analyzed extract, whose relationship to assay-associated antioxidant responses appears context-dependent and shaped by interactions among extraction selectivity, extract composition, and analytical methodology.

### 3.4. Toward a Context-Dependent Interpretation of Antioxidant Activity

The results of this study suggest that antioxidant-associated responses in complex plant extracts may not be adequately interpreted using a universal or one-dimensional analytical model. Instead, the obtained data support the concept that the resulting antioxidant response represents a context-dependent analytical phenomenon shaped by interactions among extraction chemistry, extract chemical composition, assay selectivity, solvent polarity, and reaction environment. This interpretation is consistent with the proposed Assay–Extraction Interaction Framework (AEIF), which conceptualizes antioxidant-associated responses as emergent assay-dependent phenomena arising within a specific analytical context. Similar concerns regarding the multidimensional and assay-dependent nature of antioxidant evaluation have also been discussed in methodological studies focused on antioxidant chemistry and polyphenol-rich plant systems [14,15,20].

The majority of published studies in antioxidant research interpret antioxidant activity primarily on the basis of absolute assay outputs or TPC values, frequently assuming direct analytical comparability among results. However, the findings of the present study suggest that individual assay systems may selectively respond to different antioxidant fractions and that extraction strategy may influence which chemical groups become analytically emphasized. Consequently, differences among published studies may reflect not only biological variability of analyzed materials, but also methodological heterogeneity introduced by extraction conditions, assay chemistry, and analytical design. Comparable limitations in cross-study comparability have also been highlighted in studies focused on antioxidant assay standardization and methodological interpretation [23,24,27].

This issue may be particularly relevant in complex plant matrices containing diverse phenolic compounds, prenylated flavonoids, α-bitter acids, and other redox-active constituents differing in polarity, molecular structure, solubility, and reaction kinetics [10,21,22,23]. Within such systems, antioxidant assays may not represent identical analytical dimensions of antioxidant behavior, but rather selectively emphasize different analytically detectable antioxidant fractions depending on assay chemistry, extraction selectivity, and reaction conditions. Previous studies have similarly demonstrated that assay systems differ in their responsiveness toward structurally distinct antioxidant groups and that antioxidant rankings may vary considerably depending on the analytical methodology employed [18].

Comparable issues related to limited comparability among antioxidant assay systems have also been discussed in methodological studies focused on food antioxidant systems and antioxidant analysis, where differences in assay responsiveness, extraction selectivity, and antioxidant interpretation were observed across individual analytical platforms [3,14,15,20]. Collectively, these studies suggest that individual assay systems may not represent identical aspects of antioxidant-associated behavior and that direct comparison of antioxidant outputs across studies may contribute to oversimplified interpretations of complex plant extracts.

An additional source of variability is the use of different extraction protocols, solvent systems, assay conditions, calibration standards, and data normalization approaches, which may generate systematically different outcomes even among chemically comparable extracts [15,17,24,27]. Importantly, the present study further highlights that meaningful cross-study interpretation of antioxidant datasets may benefit from prior analytical harmonization of response direction, calibration context, and assay-associated reporting systems before multivariate comparison is performed. This aspect was particularly relevant for IC50-based datasets, whose inverse transformation was necessary to establish consistent directional interpretation across analytically heterogeneous antioxidant outputs. Therefore, a substantial proportion of variability reported in antioxidant literature may reflect not only biological variability of analyzed materials, but also analytical heterogeneity arising from differences in assay selectivity, extraction chemistry, methodological design, and reporting structure.

The findings of this study further suggest that ranking-based approaches, in which extracts are primarily evaluated according to the highest antioxidant activity or TPC values, may oversimplify the multidimensional nature of antioxidant systems. Elevated assay-associated responses do not necessarily indicate broader antioxidant behavior across analytical systems, but may instead reflect preferential interactions between specific antioxidant fractions and the applied analytical methodology. Similar concerns regarding overinterpretation of absolute antioxidant values and direct translation of assay outputs into broader antioxidant relevance have also been raised in recent antioxidant methodology studies [20,26].

**Figure 6:**
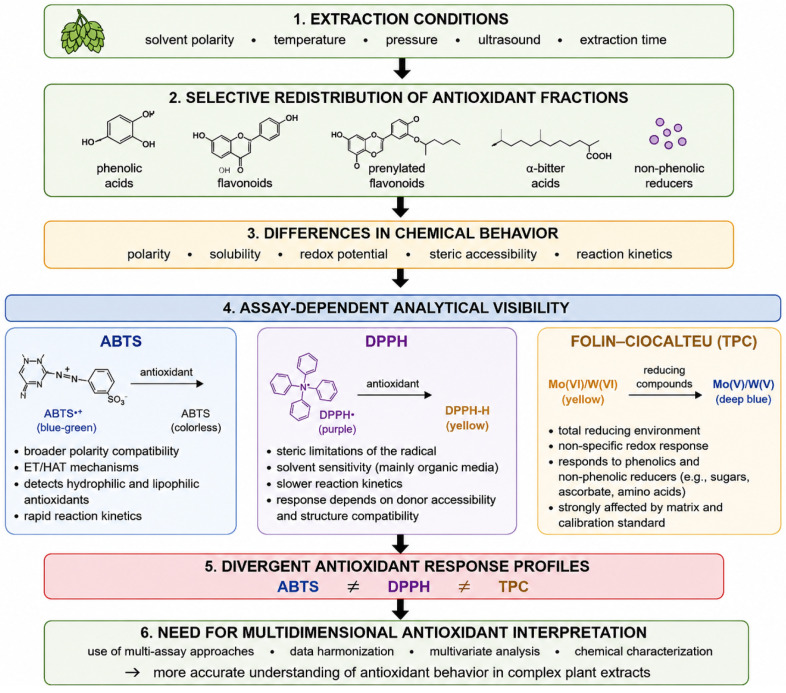
Interpretative visual summary illustrating how extraction conditions and assay chemistry may contribute to differential analytical detectability of antioxidant fractions in hop extracts. Differences in polarity, solubility, redox properties, steric accessibility, and reaction kinetics may influence assay-associated responses in ABTS, DPPH, and Folin–Ciocalteu systems, thereby contributing to divergent antioxidant response patterns and limited direct cross-study comparability.

Within this context, harmonization of analytical datasets, normalization of assay outputs, and implementation of multivariate analytical approaches may represent important steps toward improved interpretation of antioxidant literature and enhanced cross-study comparability (Figure 6). Consequently, the present study does not merely compare extraction techniques or antioxidant assay systems, but rather proposes a broader interpretative framework for evaluating antioxidant-associated responses in chemically heterogeneous plant extracts. While Figure 1 conceptually introduces the Assay–Extraction Interaction Framework (AEIF), Figure 6 provides an illustrative representation of how extraction context and assay chemistry may collectively influence the analytical visibility of antioxidant fractions and contribute to assay-associated response variability.

### 3.5. Limitations of Cross-Study Antioxidant Data Comparability

Although the present study provides an interpretative perspective on assay-associated variability in antioxidant responses, several methodological limitations associated with harmonization of data derived from different experimental systems should be considered when interpreting the results.

One of the major limitations of antioxidant literature is the high heterogeneity of analytical protocols used for determination of antioxidant responses and TPC values. Individual studies employ different extraction solvents, extraction ratios, extraction times, reaction conditions, calibration standards, assay endpoints, and reporting systems, which substantially complicate direct comparability among datasets [24,27,28,29]. Similar concerns regarding methodological inconsistency and limited cross-study comparability have also been highlighted in previous studies focused on antioxidant assay standardization and interpretation of antioxidant datasets [20,23].

An additional source of variability was associated with the use of different calibration systems for TPC determination. Although the majority of included studies reported TPC as gallic acid equivalents (GAE), some published works employed alternative calibration systems, such as ferulic acid equivalents (FAE), catechin equivalents, or tannic acid equivalents, which may generate systematically different outcomes and further complicate inter-study comparability. For this reason, FAE-derived datasets were interpreted only qualitatively in the present study and were not included in the primary statistical analysis. Similar issues related to calibration-dependent variability in antioxidant and TPC analyses have also been discussed in methodological studies focused on polyphenol quantification and antioxidant assay interpretation [25,26].

Another limitation was the restricted availability of detailed raw data in several published studies. Some studies reported only mean antioxidant response values without access to individual measurements, standard deviations, reaction kinetics, or detailed assay conditions. In some cases, critical experimental parameters such as solvent-to-solid ratios, reaction times, or calibration procedures were incompletely described. These limitations restricted deeper dataset harmonization and may partially contribute to variability observed in the cross-study analysis. Similar limitations associated with incomplete methodological reporting have also been recognized as major obstacles in antioxidant meta-analytical and comparative studies [28,29].

Importantly, normalization and harmonization applied in the present study were not intended to establish absolute equivalence among independently generated antioxidant datasets, but rather to enable relative exploratory interpretation of assay-associated response patterns across analytically heterogeneous studies. The applied normalization strategy therefore served primarily as an interpretative and comparative analytical tool facilitating multivariate visualization, trend identification, and hypothesis generation within heterogeneous antioxidant datasets.

When interpreting the results, it is also necessary to consider that the analyzed assay systems may not represent identical analytical dimensions of antioxidant-associated behavior and that responses of complex plant extracts cannot be reduced to a single analytical parameter [14,15,18,30]. The resulting antioxidant-associated responses may instead reflect interactions among extraction selectivity, extract chemical composition, assay chemistry, solvent compatibility, and reaction kinetics rather than a universal measure of antioxidant potential. Future studies should validate whether the observed assay divergence persists within experimentally controlled datasets.

An additional consideration is that the present study was designed primarily to evaluate assay-associated response patterns across analytically heterogeneous datasets rather than to directly resolve compound-specific mechanisms underlying the observed divergence between assay systems. Although the observed response patterns are chemically plausible and supported by literature describing differing reactivity of phenolic acids, prenylated flavonoids, α-bitter acids, and other phytochemical groups, direct compound-resolved validation using experimentally controlled extract systems remains warranted. Future research integrating controlled extraction experiments with detailed chemical profiling may help clarify how extraction selectivity influences assay-associated antioxidant responses and whether the observed divergence persists within experimentally standardized datasets.

Despite these limitations, the integration of literature-derived datasets, original experimental data, and multivariate analytical approaches enabled identification of reproducible assay-associated response patterns across analytically heterogeneous datasets. The findings of this study therefore highlight the need for greater methodological harmonization, improved reporting transparency, and more context-aware interpretation of antioxidant datasets in future antioxidant research, while simultaneously supporting interpretation of antioxidant-associated responses as chemically and analytically context-dependent phenomena.

### 3.6. Future Perspectives: Toward a Multi-Assay Antioxidant Response Profiling Framework

The findings of this study suggest that current approaches to evaluating antioxidant-associated responses in plant extracts may be overly reductionist when based on a single analytical assay or a single numerical parameter. The observed divergence between ABTS-, DPPH-associated responses and TPC values indicates that different assay systems may selectively respond to distinct antioxidant fractions and therefore provide differing analytical perspectives on chemically heterogeneous extracts. Similar limitations of single-assay antioxidant interpretation and assay-associated variability have been discussed in previous studies focused on antioxidant methodology and complex plant antioxidant systems [2,4,8,18,20]. These observations further support interpretation of antioxidant-associated responses within the context of assay chemistry, extraction selectivity, solvent compatibility, and extract chemical composition rather than as universally comparable analytical outputs.

The present study additionally suggests that extraction solvents and extraction techniques may influence not only extraction efficiency, but also the analytical detectability of antioxidant fractions subsequently captured by different assay systems. Similar solvent- and extraction-dependent shifts in phenolic composition and antioxidant-associated responses have previously been reported in hop and plant polyphenol extraction studies [6,7,9,10,17]. Such variability may contribute to the limited cross-study comparability frequently observed in antioxidant literature. In this context, the obtained findings support consideration of multidimensional analytical models integrating multiple complementary analytical perspectives rather than reliance on single-assay ranking approaches.

Future research may therefore benefit from development of standardized multi-assay antioxidant response profiling frameworks combining multiple antioxidant assays, harmonized data processing strategies, and multivariate analytical approaches. Such frameworks may facilitate interpretation of antioxidant-associated responses as multidimensional assay-dependent profiles reflecting both chemical heterogeneity and assay-selective analytical visibility within complex plant extract systems. Similar recommendations emphasizing multi-method analytical approaches, chemometric integration, and improved methodological harmonization have also been proposed in previous antioxidant methodology studies [2,8,28,29].

Potential components of such frameworks may include combined application of multiple antioxidant assays, normalized analytical outputs, PCA-based multivariate analysis, correlation modeling, and advanced chemical characterization of extracts, including targeted profiling of prenylated flavonoids, phenolic acids, and α-bitter acids. Integration of these approaches may improve interpretation and cross-study comparability of antioxidant-associated responses in chemically heterogeneous biological matrices [21,22,23]. Future studies may additionally benefit from approaches accounting for study-level clustering and methodological heterogeneity, including mixed-effects or hierarchical analytical models, particularly when integrating analytically heterogeneous datasets derived from independent studies.

Overall, the findings of this study support the need for future antioxidant research to move beyond isolated single-assay interpretations toward multidimensional analytical frameworks capable of more accurately capturing assay selectivity and chemical heterogeneity within plant-derived antioxidant systems.

## 4. Materials and Methods

### 4.1. Literature Search, Dataset Selection, and Data Collection

A structured literature search was performed between January and March 2026 to identify studies reporting antioxidant-associated responses in hop (*Humulus lupulus* L.) extracts obtained using different extraction systems and antioxidant assays. Scientific databases including Web of Science, Scopus, and ScienceDirect were systematically searched using combinations of keywords related to hop extraction, antioxidant activity, ABTS, DPPH, total phenolic content (TPC), polyphenols, solvent extraction, and extraction techniques. Google Scholar was additionally used as a supplementary source for manual cross-referencing of relevant publications. The final literature search was conducted in March 2026.

The literature search focused specifically on studies reporting experimentally measurable antioxidant-associated responses obtained from hop-derived extracts under defined extraction conditions. Studies were considered eligible if they provided: (i) clearly described extraction procedures, (ii) defined extraction solvents or solvent compositions, (iii) at least one antioxidant assay output (ABTS, DPPH, or TPC), and (iv) numerical analytical data suitable for harmonization, normalization, and comparative analytical interpretation.

Studies were excluded if they: (i) lacked sufficient methodological description of extraction conditions or antioxidant assay protocols, (ii) reported only qualitative antioxidant observations without numerical analytical outputs, (iii) focused exclusively on biological assays without analytical antioxidant measurements, or (iv) presented data in non-interpretable, non-extractable, or analytically incompatible formats unsuitable for harmonization and multivariate analysis.

In total, 78 potentially relevant studies were initially identified through database searching and manual cross-referencing. Following eligibility screening, only seven literature-derived studies fulfilled the predefined inclusion criteria and provided sufficiently detailed and numerically interpretable datasets suitable for harmonization and comparative analytical evaluation. The most frequent reasons for exclusion included incomplete reporting of extraction conditions, missing numerical antioxidant outputs, incompatible analytical reporting formats, insufficient methodological transparency, or lack of extract-level antioxidant data.

For each eligible study, the following metadata were recorded: extraction method, solvent type, solvent concentration, extraction conditions, assay type, reported analytical unit, calibration standard, and response direction. Additional contextual information regarding assay conditions and reporting structure was also considered to facilitate analytical harmonization across heterogeneous datasets.

The final harmonized dataset integrated literature-derived antioxidant datasets with the original experimental data generated in this study. Only datasets reporting TPC as gallic acid equivalents (GAE) were included in the primary statistical analysis to minimize calibration-dependent variability and improve comparability among studies. Datasets reported using alternative calibration systems, including ferulic acid equivalents (FAE), catechin equivalents, or tannic acid equivalents, were interpreted qualitatively and excluded from the primary correlation and multivariate statistical analyses.

Because the included datasets originated from independently generated studies conducted under heterogeneous analytical conditions, including differences in extraction procedures, solvent systems, assay protocols, calibration approaches, and reporting formats, the harmonized dataset was used primarily for exploratory comparative interpretation of assay-associated response patterns rather than direct causal inference. The applied harmonization strategy was intended to facilitate relative comparison, trend identification, and multivariate interpretation of analytically heterogeneous antioxidant datasets.

**Figure 7:**
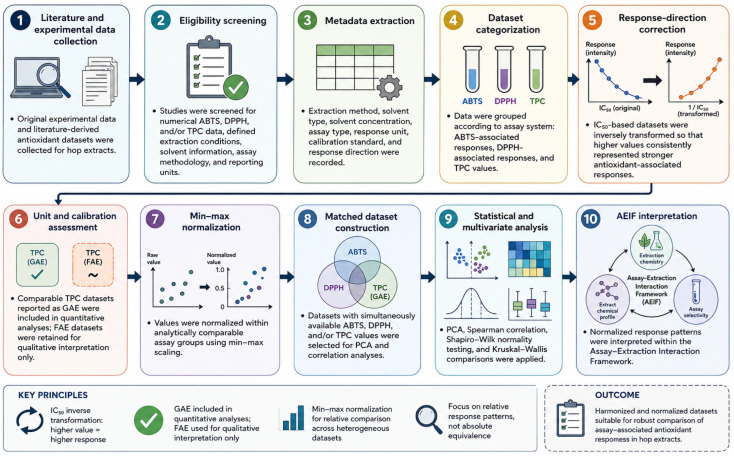
Workflow illustrating literature selection, dataset harmonization, normalization, and statistical integration of original experimental data with literature-derived antioxidant datasets. The workflow included eligibility screening, metadata extraction, assay-based categorization, response-direction correction, calibration assessment, min–max normalization, matched dataset construction, and multivariate statistical analysis. IC50-based datasets were inversely transformed prior to normalization to ensure consistent response directionality, whereas TPC datasets reported as ferulic acid equivalents (FAE) were interpreted qualitatively and excluded from the primary statistical harmonization.

The complete workflow used for dataset selection, harmonization, normalization, and statistical integration is summarized in Figure 7. The complete harmonized dataset, including literature-derived raw data, extraction metadata, assay classification, normalization parameters, and processed analytical outputs, is provided in the Supplementary Materials.

### 4.2. Data Processing, Harmonization, and Standardization

Because antioxidant-associated responses were reported using heterogeneous analytical formats across the included studies, a multistep harmonization strategy was applied prior to statistical analysis to facilitate relative comparative interpretation across analytically diverse datasets.

Initially, datasets were categorized into analytically comparable assay groups corresponding to ABTS-associated responses, DPPH-associated responses, and total phenolic content (TPC) datasets. Since antioxidant outputs were reported using different analytical formats and response directions, response-direction harmonization was performed before normalization. In datasets reported as IC50 values, inverse transformation was applied so that higher transformed values consistently represented stronger antioxidant-associated responses. This procedure enabled unified directional interpretation across heterogeneous analytical outputs while preserving relative response relationships among datasets.

Where sufficient methodological information was available, unit harmonization was additionally performed to improve analytical comparability among studies. Only TPC datasets reported as gallic acid equivalents (GAE) were included in primary quantitative statistical analyses. Datasets reported using alternative calibration systems, including ferulic acid equivalents (FAE), catechin equivalents, or tannic acid equivalents, were retained exclusively for qualitative interpretation and excluded from primary statistical harmonization to minimize calibration-dependent analytical bias. Following harmonization, min–max normalization was applied separately within analytically comparable assay groups according to the following equation:

$$x_{norm}=\frac{x-x_{min}}{x_{max}-x_{min}}$$

Normalization was performed exclusively to facilitate relative comparison of assay-associated response patterns across analytically heterogeneous datasets and was not intended to establish absolute equivalence between independently generated antioxidant values. Consequently, normalized outputs were used only for comparative statistical evaluation, multivariate visualization, and interpretation of relative assay-associated response patterns.

A total of seven literature-derived studies fulfilled the eligibility criteria and were included in the harmonized analytical framework. However, one TPC dataset reported antioxidant-associated responses using ferulic acid equivalents (FAE) and was therefore retained for qualitative interpretation only rather than primary quantitative harmonization, resulting in partial representation within multivariate statistical analyses.

Because the harmonized dataset integrated independently generated observations originating from heterogeneous analytical conditions, subsequent statistical analyses were interpreted primarily within an exploratory comparative framework rather than as evidence of direct causal relationships.

### 4.3. Experimental Extraction Procedures

Experimental hop extracts were prepared to generate chemically heterogeneous extract systems differing in extraction selectivity and solvent composition. The experimental design was structured to produce analytically diverse extract matrices suitable for comparative evaluation across multiple antioxidant assay systems.

Commercial hop pellets (*Humulus lupulus* L.) of the Magnum, Amarillo, and Galaxy cultivars harvested in 2025 were obtained from Maroma.cz (Prague, Czech Republic). Absolute methanol, absolute ethanol, and 50% (*v*/*v*) aqueous ethanol of HPLC grade (Merck KGaA, Darmstadt, Germany) were used as extraction solvents.

For all extraction procedures, 1 g of hop pellet sample was mixed with 20 mL of the corresponding extraction solvent (solid-to-liquid ratio 1:20, *w*/*v*). Maceration extraction was performed in sealed extraction tubes protected from light using aluminum foil and continuously agitated on a laboratory shaker (IKA KS 4000 i control, IKA-Werke GmbH & Co., KG, Staufen, Germany) at laboratory temperature for 24 h.

Ultrasound-assisted extraction was carried out using an ultrasonic bath (Elmasonic S 30H, Elma Schmidbauer GmbH, Singen, Germany). Samples were extracted for 30 min at 30 °C under identical solid-to-liquid conditions (1 g/20 mL). Extraction tubes were protected from direct light exposure throughout the extraction process.

Accelerated solvent extraction (ASE) was performed using a Dionex ASE 350 system (Thermo Fisher Scientific, Waltham, MA, USA) operated at a constant pressure of 10.5 MPa. ASE extraction cells were filled with 1 g of hop material mixed with 2 g of diatomaceous earth to ensure homogeneous solvent distribution and minimize channel formation during extraction. Extractions were performed at 50 °C using three consecutive static extraction cycles of 5 min each [31]. The final extraction volume was adjusted to 20 mL. Final volume adjustment was performed to ensure comparable solid-to-solvent conditions across extraction systems and facilitate relative analytical comparison.

Following extraction, all samples were centrifuged (Eppendorf 5810 R, Eppendorf AG, Hamburg, Germany) at 5000 rpm for 5 min to remove insoluble matrix components prior to analysis. The resulting supernatants were immediately subjected to ABTS radical scavenging assay, DPPH radical scavenging assay, and Folin–Ciocalteu total phenolic content (TPC) determination. All extractions and analytical measurements were performed in triplicate to ensure experimental reproducibility.

### 4.4. ABTS, DPPH, and TPC Determination

ABTS-associated antioxidant responses were determined using the ABTS radical cation decolorization assay according to a modified procedure described previously [11,32]. The ABTS radical cation (ABTS•+) was generated by reacting a 7 mM ABTS (Sigma-Aldrich, St. Louis, MO, USA) solution with 2.45 mM potassium persulfate (Sigma-Aldrich, St. Louis, MO, USA) and allowing the mixture to stand in the dark at laboratory temperature for 12–16 h prior to analysis. Before measurement, the radical solution was diluted with ethanol to obtain an absorbance of 0.70 ± 0.02 at 734 nm.

For the assay, 20 µL of extract was mixed with 980 µL of the ABTS working solution. The reaction mixture was incubated in the dark at laboratory temperature for 6 min, after which absorbance was measured at 734 nm using a UV–Vis spectrophotometer. Ethanol was used as the blank. Antioxidant-associated responses were expressed as percentage radical scavenging activity.

DPPH-associated antioxidant responses were determined using the DPPH radical scavenging assay according to previously described methodologies with minor modifications [21,25]. A freshly prepared DPPH (Sigma-Aldrich, St. Louis, MO, USA) solution in methanol was adjusted to an absorbance of approximately 0.90 ± 0.02 at 515 nm prior to analysis.

For the assay, 100 µL of extract was mixed with 3.9 mL of DPPH solution and incubated in the dark at laboratory temperature for 30 min. Absorbance was subsequently measured at 515 nm against methanol as the blank. Radical scavenging activity was expressed as percentage inhibition relative to the control solution.

Total phenolic content (TPC) was determined using the Folin–Ciocalteu method according to Blainski et al. [33,34] with minor modifications. Briefly, 100 µL of extract was mixed with 500 µL of Folin–Ciocalteu reagent (Merck KGaA, Darmstadt, Germany) diluted ten-fold with distilled water. After 5 min, 400 µL of 7.5% sodium carbonate (Merck KGaA, Darmstadt, Germany) solution was added. The reaction mixture was incubated in the dark at laboratory temperature for 30 min, after which absorbance was measured at 750 nm. Gallic acid (Sigma-Aldrich, St. Louis, MO, USA) was used as the calibration standard, and TPC values were expressed as mg gallic acid equivalents (GAE) per g of dry weight. All spectrophotometric measurements were performed in triplicate.

### 4.5. Statistical Analysis

The statistical workflow consisted of dataset harmonization, assay-specific normalization, exploratory multivariate analysis, non-parametric correlation analysis, and comparison of extraction-associated response variability across the analyzed datasets.

Principal component analysis (PCA) was performed using normalized ABTS-, DPPH-, and TPC-associated response datasets to visualize assay-associated variability and multivariate response patterns across the harmonized dataset. Because the analyzed data originated from heterogeneous analytical conditions and independently generated studies, PCA was applied as an exploratory multivariate visualization tool to identify covariance structures and response patterns rather than infer direct causal or mechanistic relationships.

Relationships among normalized antioxidant-associated response datasets were evaluated using Spearman rank correlation analysis due to the non-normal distribution observed in several assay-associated datasets. Normality was assessed using the Shapiro–Wilk test prior to correlation analysis.

Differences between extraction-associated response groups categorized according to extraction technique and solvent type were evaluated using the Kruskal–Wallis test. Non-parametric statistical approaches were selected because several datasets did not satisfy assumptions of normal distribution and analytical homogeneity.

All statistical analyses were performed using GraphPad Prism version 10.0 (GraphPad Software, San Diego, CA, USA) and Microsoft Excel (Microsoft Corporation, Redmond, WA, USA). Statistical significance was considered at *p* < 0.05.

## 5. Conclusions

The present findings do not question the analytical usefulness of established antioxidant assays, but rather emphasize the importance of context-aware interpretation of assay-associated responses. The present study provides an interpretative perspective on assay-dependent variability in antioxidant-associated responses within complex hop extracts and highlights limitations of one-dimensional approaches to antioxidant evaluation. The obtained findings suggest that ABTS-, DPPH-associated responses, and TPC values did not exhibit consistent relationships across the analyzed extraction conditions, indicating that individual assay systems may not represent identical analytical dimensions of antioxidant behavior. The results further suggest that extraction solvents and extraction techniques may influence not only extraction efficiency, but also the analytical detectability of antioxidant fractions subsequently captured by individual assay systems. Consequently, antioxidant-associated responses may not represent a universal inherent property of the extract, but rather context-dependent analytical outcomes shaped by interactions among extraction chemistry, extract composition, and assay selectivity. Within this context, the present study proposes the Assay–Extraction Interaction Framework (AEIF), which conceptualizes antioxidant-associated responses as context-dependent analytical phenomena arising within a specific analytical environment. The proposed framework further contributes to interpretation of the high variability and limited comparability frequently observed in antioxidant literature. Overall, the findings support the need for multidimensional analytical approaches integrating multiple assay systems, data harmonization, and chemometric tools to improve interpretation of antioxidant-associated responses in chemically heterogeneous biological matrices. Beyond hop extracts, the proposed framework may also provide a broader conceptual basis for interpretation of antioxidant responses in complex plant-derived systems.

## Figures and Tables

**Table 1:** Mechanistic characteristics, analytical selectivity, and principal limitations of ABTS, DPPH, and Folin–Ciocalteu systems used for antioxidant response evaluation in complex plant extracts.

Assay System	Main Analytical Principle	Dominant Chemical Interpretation	Preferential Analytical Visibility	Key Limitations	Relevance to This Study
ABTS	Reduction of the ABTS radical cation	Electron transfer and hydrogen atom transfer reactions	Broad spectrum of hydrophilic and lipophilic antioxidants	Strongly affected by reaction medium, reaction time, and assay conditions	Captured antioxidant fractions that differed from those detected by DPPH, supporting assay-dependent response behavior
DPPH	Reduction of the stable DPPH radical	Mainly radical scavenging response influenced by donor availability and steric accessibility	Compounds compatible with the reaction solvent and accessible to the sterically hindered radical	Limited solubility in aqueous systems, steric constraints, slower reaction kinetics	Showed divergent responses compared with ABTS, indicating selective analytical visibility of different antioxidant fractions
Folin–Ciocalteu/TPC	Reduction of phosphomolybdic–phosphotungstic reagent under alkaline conditions	Total reducing capacity rather than selective phenolic quantification	Phenolics and other reducing compounds, including non-phenolic reducers	Non-specific response; affected by calibration standard, reducing sugars, amino acids, ascorbate, and other reductants	TPC did not uniformly predict ABTS- or DPPH-associated responses, supporting the limitation of TPC as a standalone antioxidant marker

## Data Availability

All data supporting the findings of this study are included in the manuscript and its Supplementary Materials.

## Supplementary Materials

The following supporting information can be downloaded at: https://www.mdpi.com/article/10.3390/molecules31122066/s1, Supplementary materials associated with this article include harmonized datasets, methodological details, complementary multivariate visualizations, and complete raw experimental data supporting the statistical analyses and mechanistic interpretation presented in the manuscript. Table S1: Harmonized antioxidant response dataset used for multivariate and correlation analyses; Table S2. Experimental extraction conditions and analytical workflow used in this study; Figure S1. Heatmap visualization of normalized assay-associated antioxidant response profiles across experimental and literature-derived hop extract datasets.

## Abbreviations

The following abbreviations are used in this manuscript:

AEIF	Assay–Extraction Interaction Framework
ABTS	2,2′-Azino-bis(3-ethylbenzothiazoline-6-sulfonic acid)
DPPH	2,2-Diphenyl-1-picrylhydrazyl
TPC	Total Phenolic Content
PCA	Principal Component Analysis
GAE	Gallic Acid Equivalents
FAE	Ferulic Acid Equivalents
TEAC	Trolox Equivalent Antioxidant Capacity
ASE	Accelerated Solvent Extraction
ET	Electron Transfer
HAT	Hydrogen Atom Transfer
IC50	Half Maximal Inhibitory Concentration
TE	Trolox Equivalents
UAE	Ultrasound-Assisted Extraction
