# Supplementary material for: Assay-Dependent Variability of Antioxidant Responses in Hop Extracts: Implications for Cross-Study Comparability and Antioxidant Interpretation
Source: Molecules. 2026 Jun 12;31(12):2066. doi: 10.3390/molecules31122066 (PMC13304896; doi:10.3390/molecules31122066)
Supplement: Supplementary file 1 [file molecules-31-02066-s001.zip › molecules-4347257-supplementary.pdf]

Supplementary materials

The Supplementary Materials provide complete supporting datasets, methodological details, harmonized analytical outputs, and complementary multivariate visualizations associated with the mechanistic interpretation of assay-dependent antioxidant responses discussed in the main manuscript. To improve analytical transparency, reproducibility, and interpretability of cross-study antioxidant datasets, complete raw experimental data and processed datasets used for statistical analyses are additionally provided.

Supplementary Table S1 summarizes the harmonized experimental and literature-derived datasets included in multivariate statistical analyses, including extraction conditions, assay systems, normalization procedures, analytical transformation strategy, and subsequent statistical application. Since antioxidant activity values were originally reported using heterogeneous analytical formats across individual studies, harmonization procedures were applied to enable comparative multivariate interpretation while preserving assay-associated response directionality.

Supplementary Table S2 provides a detailed overview of extraction procedures, analytical workflow, assay conditions, centrifugation parameters, normalization procedures, and statistical analyses applied throughout the study to facilitate methodological reproducibility.

Supplementary Figure S1 presents the heatmap visualization of normalized antioxidant response profiles across different extraction systems, solvents, and assay conditions, illustrating extraction-dependent redistribution of analytically detectable antioxidant fractions and assay-selective response divergence patterns supporting the multidimensional nature of antioxidant behavior in complex hop extract systems.

Complete raw datasets associated with ABTS, DPPH, TPC, extraction conditions, normalization procedures, PCA input matrices, and statistical processing are provided as Supplementary Data File S1 to ensure full analytical transparency, facilitate reproducibility, and support independent secondary interpretation of the presented multivariate analyses.

Table S1. Harmonized antioxidant response dataset used for multivariate and correlation analyses

Study / Dataset Source	Sample Type	Extraction Method	Solvent System	Assay System	Original Reported Unit	Harmonized Analytical Interpretation	Data Transformation Applied	Normalized Dataset Usage
Experimental dataset (this study)	Hop extracts (Amarillo, Magnum, Galaxy)	Maceration	50% ethanol	ABTS	TE equivalents	ABTS-associated antioxidant response	Min–max normalization	PCA, correlations, Kruskal–Wallis
Experimental dataset (this study)	Hop extracts (Amarillo, Magnum, Galaxy)	Maceration	Absolute ethanol	ABTS	TE equivalents	ABTS-associated antioxidant response	Min–max normalization	PCA, correlations, Kruskal–Wallis
Experimental dataset (this study)	Hop extracts (Amarillo, Magnum, Galaxy)	Ultrasound extraction	Methanol	DPPH	% inhibition	DPPH-associated antioxidant response	Min–max normalization	PCA, correlations, Kruskal–Wallis
Experimental dataset (this study)	Hop extracts (Amarillo, Magnum, Galaxy)	ASE 350	50% ethanol	ABTS	TE equivalents	ABTS-associated antioxidant response	Min–max normalization	PCA, correlations, Kruskal–Wallis
Experimental dataset (this study)	Hop extracts (Amarillo, Magnum, Galaxy)	ASE 350	Methanol	DPPH	% inhibition	DPPH-associated antioxidant response	Min–max normalization	PCA, correlations, Kruskal–Wallis

Study / Dataset Source	Sample Type	Extraction Method	Solvent System	Assay System	Original Reported Unit	Harmonized Analytical Interpretation	Data Transformation Applied	Normalized Dataset Usage
[1]	Hop extracts	Solvent extraction	Ethanol-based systems	TPC	GAE	Total reducing capacity	Included without inversion	PCA, correlations
[4]	Hop products and standards	Multiple extraction systems	Mixed solvents	ABTS / DPPH	IC50 / TEAC	Assay-associated antioxidant response	IC50 inversion + normalization	PCA, correlations
[7]	Hop strobile extracts	Solvent extraction	Ethanol / methanol	DPPH / TPC	% inhibition / GAE	Assay-associated antioxidant response and TPC	Min–max normalization	PCA, correlations
[9]	Hop extracts	Soxhlet / ASE extraction	Ethanol systems	ABTS / DPPH	IC50 / TE equivalents	Assay-associated antioxidant response	IC50 inversion + normalization	PCA, Kruskal–Wallis
[2]	Literature-derived hop datasets	Review dataset	Multiple solvent systems	ABTS / DPPH / TPC	Mixed reporting systems	Cross-study antioxidant response variability	Harmonized qualitative comparison	Discussion support
[5]	Hop phenolic extracts	Solvent extraction	Ethanol	ABTS / DPPH	TE equivalents	Assay-associated antioxidant response	Min–max normalization	Comparative interpretation

Table S1. Harmonized experimental and literature-derived antioxidant response datasets included in multivariate statistical analyses. Antioxidant-associated responses reported in heterogeneous analytical formats were categorized into assay-associated datasets (ABTS, DPPH, and TPC) and harmonized to enable comparative multivariate interpretation across analytically diverse studies. Datasets reported as IC50 values were inversely transformed prior to min–max normalization to ensure unified response directionality, where higher values consistently represented stronger antioxidant-associated responses. TPC datasets reported as gallic acid equivalents (GAE) were included in primary quantitative analyses, whereas datasets reported using alternative calibration systems were interpreted qualitatively and excluded from primary statistical harmonization. Abbreviations: TE, Trolox equivalents; TEAC, Trolox equivalent antioxidant capacity; GAE, gallic acid equivalents; ASE, accelerated solvent extraction; PCA, principal component analysis; IC50, half maximal inhibitory concentration.

Table S2. Experimental extraction conditions and analytical workflow used in this study

Experimental Step	Experimental Conditions	Analytical Purpose
Plant material	Lyophilized hop samples (Amarillo, Magnum, Galaxy; harvest 2025)	Generation of chemically heterogeneous extract systems
Maceration extraction	1 g sample + 20 mL solvent; 24 h; laboratory temperature; orbital shaking	Diffusion-driven extraction model
Ultrasound extraction	1 g sample + 20 mL solvent; 30 min; 30 °C	Mild cavitation-assisted extraction
ASE 350 extraction	1 g sample; 50 °C; 25 min; 3 extraction cycles, static time 5 min; final volume 20 ml	Pressure- and temperature-assisted extraction
Solvent systems	50% ethanol, absolute ethanol, methanol	Evaluation of solvent-dependent extraction selectivity
Centrifugation	5,000 rpm; 5 min	Removal of insoluble matrix components
TPC analysis	Folin–Ciocalteu assay; 750 nm	Evaluation of total reducing capacity
ABTS analysis	$ABTS^{+}$• radical cation assay; 734 nm	Evaluation of ABTS-associated antioxidant response
DPPH analysis	DPPH radical scavenging assay; 515 nm	Evaluation of DPPH-associated antioxidant response

Experimental Step	Experimental Conditions	Analytical Purpose
Data harmonization	IC50 inversion; assay-specific min–max normalization	Cross-study comparability of analytically heterogeneous datasets
Statistical analysis	Spearman correlation, PCA, Kruskal–Wallis	Identification of assay-dependent response divergence patterns

Table S2. Overview of extraction conditions, analytical assays, data harmonization procedures, and multivariate statistical workflow applied for evaluation of assay-dependent antioxidant responses in hop extracts. Experimental conditions were intentionally selected to generate chemically heterogeneous extract systems suitable for mechanistic interpretation of assay-selective antioxidant response behavior.

Figure S1. Heatmap visualization of normalized assay-associated antioxidant response profiles across experimental and literature-derived hop extract datasets.

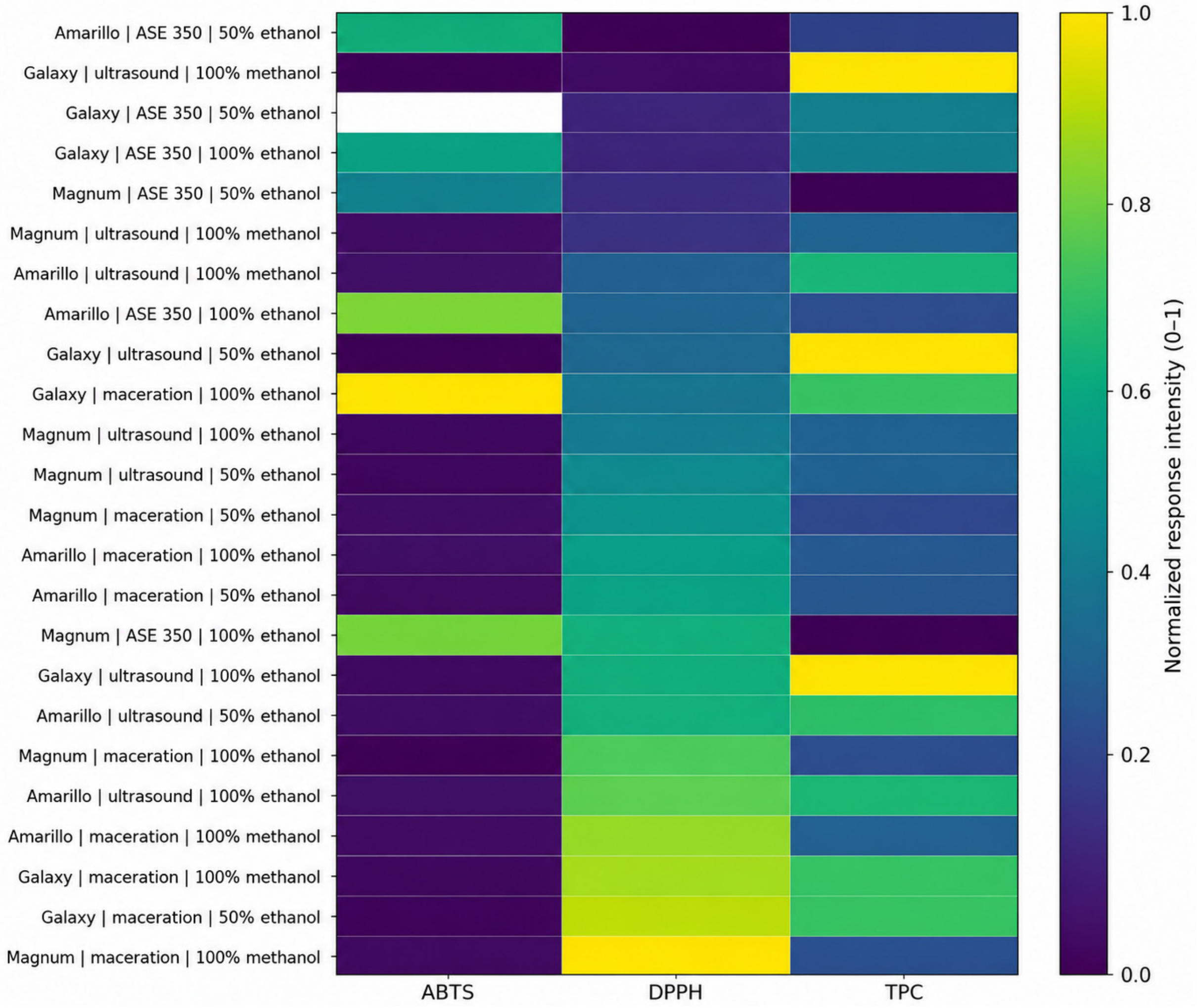

Figure S1. Heatmap visualization of normalized ABTS-, DPPH-associated responses and total phenolic content (TPC) values across different extraction systems, solvent conditions, and hop extract datasets. Color intensity represents normalized response values (0–1), illustrating variability in assay-associated antioxidant responses across the analyzed extracts. Distinct response distributions reflect heterogeneity among extraction systems and solvent conditions. The Galaxy/ASE 350/50% ethanol extract was excluded from heatmap visualization and downstream multivariate normalization because the ABTS-associated response exceeded the analytical measurement range (over range), preventing reliable inclusion in comparative normalization procedures.
